# The Posterior Parietal Cortex Is Involved in Gait Adaptation: A Bilateral Transcranial Direct Current Stimulation Study

**DOI:** 10.3389/fnhum.2020.581026

**Published:** 2020-11-05

**Authors:** David R. Young, Pranav J. Parikh, Charles S. Layne

**Affiliations:** ^1^Department of Health and Human Performance, Center for Neuromotor and Biomechanics Research, College of Liberal Arts and Social Sciences, University of Houston, Houston, TX, United States; ^2^Center for Neuro-Engineering and Cognitive Science, Cullen College of Engineering, University of Houston, Houston, TX, United States

**Keywords:** gait, adaptation, tDCS, posterior parietal cortex (PPC), split-belt walking, sensory integration

## Abstract

Gait is one of the fundamental behaviors we use to interact with the world. The functionality of the locomotor system is thus related to enriching interactions with our environment. The posterior parietal cortex (PPC) has been found to contribute to motor adaptation during both visuomotor and postural adaptation tasks. Additionally, structural or functional deficits of the PPC lead to impairments in gaits such as shortened steps and increased step width. Based on the aforementioned roles of the PPC, and the importance of gait adaptability, the current investigation sought to identify the role of the PPC in gait adaptation. To achieve this, we performed transcranial direct current stimulation (tDCS) over the bilateral PPC before performing a split-belt treadmill gait adaptation paradigm. We used three stimulation conditions in a within-subject design. tDCS was administered in a randomized and double-blinded order. Following each stimulation session, subjects first performed baseline walking with both belts running at the same speed. Then, subjects walked for 15 min on an uncoupled treadmill, with the belts being driven at a 3:1 speed ratio. Last, they returned to normal (i.e., tied-belt) walking for 5 min. Results from 15 young and healthy subjects identified that subjects required more steps to adapt to split-belt walking following the suppression of the left hemisphere PPC, contralateral to the fast belt. Furthermore, while suppression of the left hemisphere PPC did not increase the number of steps required to re-adapt to tied-belt walking, this condition did lead to increased magnitude of after-effects. Together, these findings indicate that the PPC is involved in locomotor adaptation. These results support previous literature regarding the upper body or postural adaptation and extend these findings to the realm of gait. Results highlight the PPC as a potential target for neurorehabilitation designed to improve gait adaptability.

## Introduction

Walking is among the most important ways that people interact with their environment. Therefore, locomotor dysfunctions lead to decreases in meaningful and enriching interactions, decreasing quality of life. Effective gait requires an ability to adapt one’s motions to changes in their environment. Previous studies have identified those gait parameters such as step length, step time, and double support time adapt over time to changes in the environment or task (Reisman et al., 2005). These parameters also exhibit after-effects, which is a reversal in the direction of error once the environmental perturbation is removed. After-effects confirm that adaptation is not a simple reaction, but a recalibration of the internal representation of the environment (Shadmehr et al., 2010).

Evidence suggests that adaptability is related to the motor plasticity required for successful rehabilitation following a stroke (Bastian, 2008). Even beyond diagnostics, stroke patients have exhibited improved upper body motor function following adaptation paradigms. In some cases, these improvements have lasted for five or more days from a single exposure to the adaptation paradigm (Rossetti et al., 1998). Repeated exposures to environmental perturbations, leading to faster adaptation rates, may be related to plasticity and maybe a tool to promote learning in clinical populations (Bastian, 2008). Systematic utilization of adaptation paradigms has also been suggested to improve general adaptability in non-clinical populations, such as those preparing for spaceflight. This may allow for better performance and re-integration into normal life upon returning to earth (Seidler, 2010; Bloomberg et al., 2015).

Locomotor adaptation, like all motor adaptation, requires plasticity of the internal representation of one’s position and movement within the environment (Gurfinkel et al., 1995). Because the posterior parietal cortex (PPC) is at least partially responsible for the creation and maintenance of the internal representation, the role of the PPC in motor adaptation is of interest. In their 2011 study, Mutha et al. (2011) compared visuomotor adaptation in those with unilateral lesions of the left or right parietal cortex. Patients with right hemisphere damage exhibited normal adaptation and after-effects, while those with left hemisphere damage exhibited impaired adaptation and decreased or abolished after-effects. The group suggested that these results indicate that left parietal regions are primarily responsible for visuomotor adaptation (Mutha et al., 2011). These results have been supported by fMRI studies (Crottaz-Herbette et al., 2014). On-line integration of sensory feedback is a likely mechanism for the process in which the PPC is involved in motor adaptation (Gréa et al., 2002).

A recent study by our group showed that the PPC is also involved in postural adaptation (Young et al., 2020). We found that bilateral transcranial direct current stimulation (tDCS) stimulation of PPC decreased adaptation to an incline-intervention when compared with sham stimulation. Because it is apparent that the PPC is involved in visuomotor and postural adaptation, we sought to identify whether neuromodulation of the PPC may affect gait adaptation as well. The theoretical justifications were reinforced based on findings that PPC function is related to gait performance in several populations (Bartels and Leenders, 2008; Rosano et al., 2008; Rubino et al., 2014). To the best of our knowledge, there has not been an investigation into the role of the PPC in locomotor adaptation. Previously, Jayaram et al. (2012) applied tDCS to the cerebellum during a split-belt adaptation paradigm. Performing tDCS on the ipsilateral hemisphere of the fast belt, the group identified that anodal stimulation expedited adaptation while cathodal stimulation delayed adaptation (Jayaram et al., 2012). Based on these findings, it is of interest to identify if there is an effect of non-invasive brain stimulation of the PPC on split-belt adaptation. During split-belt walking, subjects walk on a specialized treadmill, comprised of two separate belts. The subject walks with one foot on each belt, and these belts are capable of operating at two different speeds (Dietz et al., 1994). We performed split-belt adaptation paradigms after bilateral tDCS, which injects low-intensity current, flowing from anodal to the cathodal electrode(s), and results in slight alterations in the excitability of underlying cortical tissue (Lefaucheur and Wendling, 2019). To study the role of the PPC in gait adaptation, subjects were provided bilateral tDCS stimulation of the PPC. We then sought to compare stimulation conditions on measures of gait adaptation. Based on our previous experiment, we hypothesized that active tDCS would inhibit sensory error-based adaptation of step length symmetry ratios as well as delay re-adaptation to normal walking (Young et al., 2020).

## Materials and Methods

### Subjects

Subjects were recruited from the university community and provided their written informed consent following the Helsinki Declaration. All study materials including the informed consent were previously approved by the University of Houston institutional review board for experimental studies. Eligible subjects were between the ages of 18–35, able to stand and walk without assistance for a minimum of 30 min, had no history of neurological or musculoskeletal dysfunction that could inhibit gait, and had no known contraindications to tDCS stimulation such as metallic implants, history of seizures or brain damage (Datta et al., 2010).

### Gait Adaptation Protocol

**Figure 1:**
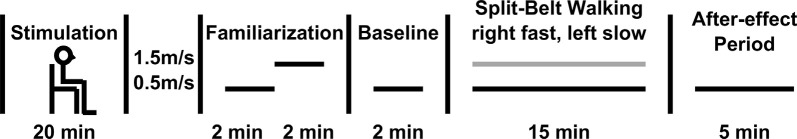
Experimental paradigm. Stimulation occurred before the gait adaptation paradigm. Gait parameters were recorded during the baseline, split-belt walking, and after-effect periods.

Following each of the three stimulation bouts, subjects performed identical split-belt adaptation paradigms. Subjects walked on a split-belt treadmill capable of independent speed control for each foot (Bertec, Columbus, OH, USA). For each session, subjects performed five phases of treadmill walking (Figure 1). Each session was initiated by 2 min of walking at a speed of 0.5 m/s and 2 min of walking at 1.5 m/s designed to familiarize the subject with walking on a treadmill. Next, subjects walked for another 2 min at 0.5 m/s to identify baseline gait characteristics. Then, subjects underwent 15 min of split-belt walking, wherein the left belt remained set to 0.5 m/s while the right belt was set to 1.5 m/s. Last, subjects underwent a 5-min after-effect period where the belts were re-tied at 0.5 m/s. A 3:1 speed ratio and 15 min period of split-belt walking are commonly utilized in the literature, including a previous study of the effects of tDCS on gait adaptation (Jayaram et al., 2012; Vasudevan et al., 2017; Yokoyama et al., 2018). Data were collected during the baseline, split-belt walking, and after-effect period phases. Sessions were separated by a minimum of 48 h.

### Brain Stimulation

**Figure 2:**
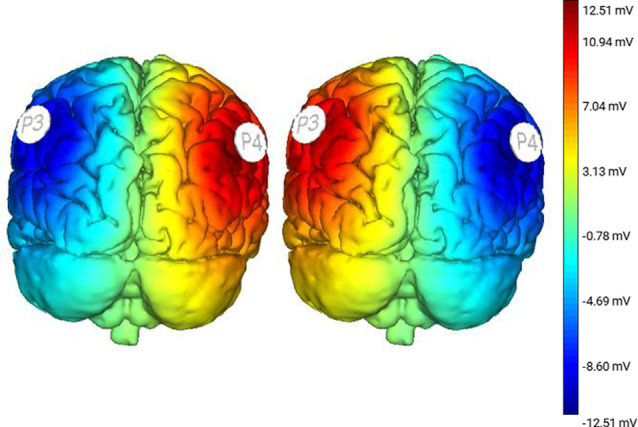
Modeling derived from protocol illustrating changes in cortical excitability following Right Anodal-Left Cathodal (RA-LC; left image) and Right Cathodal-Left Anodal (RC-LA; right image) stimulation.

Subjects were administered three conditions of non-invasive tDCS stimulation. Two active conditions were applied: Right Anodal-Left Cathodal (RA-LC), designed to slightly depolarize the right hemisphere while hyperpolarizing the left, and Right Cathodal-Left Anodal (RC-LA), designed to do the opposite. For instance, in the RA-LC condition, subjects received anodal and cathodal tDCS over PPC in the hemisphere ipsilateral and contralateral to the right (fast) leg, respectively. For both active conditions, stimulation was applied at 1.5 mA for 20 min (Figure 2). The final condition was Sham, during which current was ramped up for 30 s before being ramped down. This condition was designed to provide the sensations of active stimulation on the scalp, without altering cortical excitability. For each condition, saline-soaked 25 cm^2^ sponges were placed at locations P3 and P4 using the international 10-20 system (Homan et al., 1987). tDCS stimulation was performed using an eight-channel Starstim tDCS Device (Neuroelectrics, Spain). Stimulation conditions were administered in random order and were double-blinded to the participant as well as the administrator.

### Instrumentation and Data Processing

Subjects were outfitted with reflective markers on the heel and great toe of each foot. A 12-camera motion capture system was used to collect marker trajectories from both feet at a frequency of 100 Hz (Vicon, Oxford Metrics Limited, Oxford). Kinematic data were collected during walking and processed using custom MATLAB codes (MATLAB 2019a, MathWorks, Natick, MA, USA). Marker trajectories were filtered using a second order low-pass Butterworth filter with a cut-off frequency set to 6 Hz. Using marker trajectories, step lengths (SL) for the right and left legs were calculated. We utilized SL derived from the slow and fast (i.e., left and right) legs to calculate step length symmetry:

$$Symmetry\;(SLS{)}_{n}=\frac{\left(SL\;Slow_{n}-SL\;Fast_{n}\right)}{\left(SL\;Slow_{n}+SL\;Fast_{n}\right)}$$

we compared between conditions for two primary outcomes. Both variables were calculated for each subject and were derived from step length symmetry (SLS) ratio. For the first measure, we computed the average SLS ratio for five key periods (Table 1). We quantified average SLS ratio for baseline walking (Baseline), the first five strides following the splitting of the treadmill belts (Early Split), the final five strides (Late Split), as well as the first (Early Tied) and final five (Late Tied) strides of the after-effect period. For the second measure, we used the computed SLS values to quantify the rate of adaptation by finding the time-constant (i.e., number of steps required) to achieve adaptation. Time-constant has been utilized elsewhere (Jayaram et al., 2012). Data were smoothed to a moving window of 10 strides and then fitted using the Curve Fitting Toolbox using the formula below (MATLAB 2020a MathWorks, Natick, MA, USA). The time constant was identified as 1/k. This process was repeated during the after-effect phase.

$$y=ae^{-xk}+c$$

**Table 1:** Time periods associated with a split-belt adaptation paradigm.

Time-period	Determination
Baseline	Final five strides before belt uncoupling
Early Split	First five strides at 3:1 ratio
Late Split	Final five strides at 3:1 ratio
Early Tied	First five strides after the treadmill was recoupled
Late Tied	The final five strides after the treadmill was recoupled

### Statistical Analysis

To identify the effects of tDCS stimulation on gait adaptation, a two-way (Condition × Time) repeated measures analysis of variance (RM-ANOVA) was employed to compare between conditions for the number of steps required (i.e., the time-constant) to reach adapted walking during the split-belt period and re-tied period. Next, two follow-up, one-way RM-ANOVAs were used to compare time-constant within periods and between conditions. Then, a two-way RM-ANOVA compared SLS between conditions during Early Split and Late Split. Last, a two-way RM-ANOVA compared SLS between conditions during Early Tied and Late Tied walking. In the case of significant main effect findings, Bonferroni *post hoc* corrections were employed. Hedge’s G (HG) statistics were computed as effect sizes for pairwise differences. Effect sizes derived from partial eta squared ($\eta_{\text{p}}^{2}$) were derived in cases of significant main and interaction effects. Frequently, effect sizes are being described as small (0.2–0.5), medium (0.5–08), or large (0.8 or greater; Cohen, 1988). For all analyses, significant findings were defined by an alpha value of *p* < 0.05. All statistical analysis was performed using SPSS 26 (IBM, Armonk, NY, USA). Descriptive outcomes can be observed in Table 2.

**Table 2:** Outcomes of the split-belt adaptation paradigms.

	Sham	RA-LC	RC-LA
**Time-constant (number of steps)**
Adaptation period	47.44 ± 52.02	118.18 ± 104.93	61.77 ± 60.71
After-effect period	22.76 ± 20.48	18.49 ± 20.33	17.36 ± 17.91
**Split-belt step length symmetry ratio**
Early Split	0.34 ± 0.19	0.49 ± 0.023	0.43 ± 0.21
Late Split	0.24 ± 0.04	0.22 ± 0.13	0.025 ± 0.06
**After-effect step length symmetry ratio**
Early Tied	−0.09 ± 0.19	−0.27 ± 0.24	−0.17 ± 0.31
Late Tied	−0.04 ± 0.04	−0.06 ± 0.07	−0.06 ± 0.06

## Results

### Subjects

Fifteen subjects, eight females and seven males completed the study. Subjects were aged 23.4 ± 4.2 years, were 165.6 ± 12.57 cm tall, and 77.43 ± 18.27 kg in body mass. When asked at the end of each session to identify what stimulation condition they had received, subjects guessed correctly 1.0 ± 0.78 times out of three. A McNemar-Bowker test, performed to ascertain whether actual stimulation condition had an impact on the subject’s perception, yielded insignificant results (*p* = 0.32). This demonstrated that the stimulation condition did not affect perception.

### Adaptation Protocol

**Figure 3:**
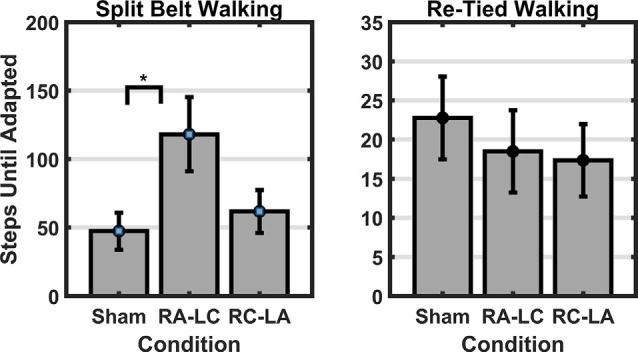
Left panel: the time-constant (i.e., number of steps) required to reach two-thirds of the adaptation curve was significantly greater following RA-LC stimulation than Sham. Right panel: no significant differences were observed in the number of steps required to adapted walking and to re-calibrate to tied-belt walking. Data reflects mean (±1 SEM). Asterisks (*) indicate statistical significance with corresponding *p*-value <0.05.

**Figure 4:**
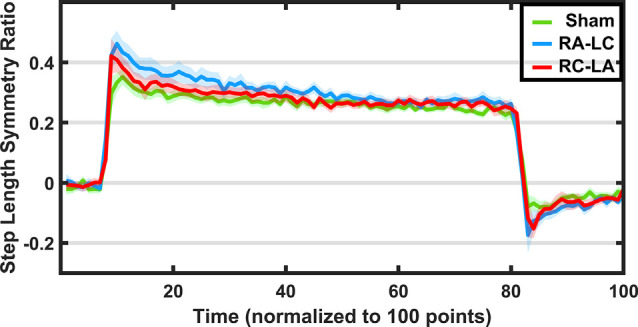
Step length symmetry (±1 SEM) throughout the adaptation protocol. Green indicates sham, blue indicates RA-LC stimulation and red indicates RC-LA stimulation. Because each subject completed each adaptation paradigm with a different total number of strides, data were normalized to 100 data points.

Results of a two-way RM-ANOVA which compared between conditions and between split-belt and re-tied walking revealed a significant main effect of condition as well as a significant interaction of condition and period, on the time-constant (i.e., number of steps to reach adapted walking; Condition: *F*_(2,13)_ = 7.92, *p* = 0.006, $\eta_{\text{p}}^{2}$ = 0.55; Time: *F*_(1,14)_ = 1.37, *p* = 0.26, $\eta_{\text{p}}^{2}$ = 0.09; Interaction: *F*_(2,13)_ = 13.68, *p* = 0.0001, $\eta_{\text{p}}^{2}$ = 0.68; Figure 3). Follow-up analyses within periods showed a significant effect of condition for the number of steps required to reach adapted walking during the split-belt period (*F*_(2,13)_ = 12.46, *p* = 0.001, $\eta_{\text{p}}^{2}$ = 0.66). Bonferroni *post hoc* analysis revealed significantly fewer steps necessary to adapt following Sham stimulation than RA-LC stimulation (*p* = 0.003, HG = 0.85), but not RC-LA stimulation (*p* = 0.29, HG = 0.54). No differences were observed between active stimulation conditions (*p* = 1). Also, no differences were observed between conditions for the number of steps required to re-adapt to normal walking following re-tying the treadmill belts (*F*_(2,13)_ = 0.39, *p* = 0.68, $\eta_{\text{p}}^{2}$ = 0.58). Time series data can be observed in Figure 4. Overall *R*^2^ values derived from the curve fitting averaged 0.61 ± 0.24 for the adaptation phase and 0.67 ± 0.22 for the after-effect phase. The grand average was 0.64 ± 0.23. There was no difference in *R*^2^ between conditions during the adaptation (*F*_(2,13)_ = 0.038, *p* = 0.963, $\eta_{\text{p}}^{2}$ = 0.006) or after-effect periods (*F*_(2,13)_ = 0.1774, *p* = 0.21, $\eta_{\text{p}}^{2}$ = 0.21).

The SLS ratio changed as subjects adapted to the split-belt paradigm (main effect of time: *F*_(1,14)_ = 31.96, *p* < 0.0001, $\eta_{\text{p}}^{2}$ = 0.69). However, there was no effect of condition on the SLS ratio at the beginning or end of the split-belt period (*F*_(2,13)_ = 0.98, *p* = 0.40, $\eta_{\text{p}}^{2}$ = 0.13). There was also no interaction effect (*F*_(2,13)_ = 2.23, *p* = 0.15, $\eta_{\text{p}}^{2}$ = 0.26). That is, across stimulation conditions, there were no differences in SLS ratio during the first five strides of split-belt walking (*F*_(2,13)_ = 1.59, *p* = 0.24, $\eta_{\text{p}}^{2}$ = 0.19) and the final five strides of split-belt walking (*F*_(2,13)_ = 0.22, *p* = 0.8, $\eta_{\text{p}}^{2}$ = 0.03).

**Figure 5:**
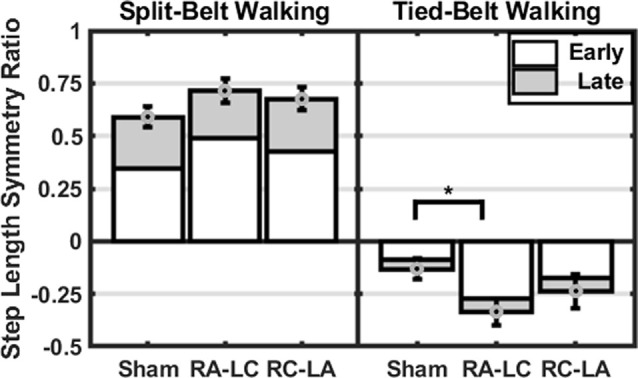
Mean step length symmetry at each time-period (±1 SEM). Asterisks (*) indicate statistical significance with corresponding *p*-value <0.05.

Importantly, the SLS ratio during the after-effect period was affected by the stimulation condition (*F*_(2,13)_ = 4.46, *p* = 0.034, $\eta_{\text{p}}^{2}$ = 0.41; Figure 5). Furthermore, a significant effect of time was found (*F*_(1,14)_ = 5.82, *p* = 0.03, $\eta_{\text{p}}^{2}$ = 0.29), but no interaction effect (*F*_(2,13)_ = 2.49, *p* = 0.12, $\eta_{\text{p}}^{2}$ = 0.28). Pairwise comparisons revealed that the RA-LC stimulation led to significantly more step asymmetry than Sham stimulation throughout the after-effect period (*p* = 0.031 HG = 0.38). No pairwise differences were observed between Sham and RC-LA stimulation (*p* = 0.52) or RA-LC and RC-LA stimulation (*p* = 0.9).

## Discussion

This investigation sought to clarify the role of the PPC in gait adaptation. Structural or functional deficits in the PPC have been described in several populations with gait dysfunctions (Bartels and Leenders, 2008; Rosano et al., 2008; Rubino et al., 2014). There is also evidence that the PPC is involved in novel or challenging gait tasks which require recalibration of the internal representation (An et al., 2019; Delval et al., 2020). Delval et al. (2020) identified that less natural walking conditions led to changes in the EEG band power of the PPC (Delval et al., 2020). An et al. (2019) identified that unpredictable trip perturbations increased cortical activity in sensorimotor and posterior parietal areas compared to normal walking (An et al., 2019). Previously, Pearson et al. have identified that PPC mechanisms are critical for working memory necessary for obstacle avoidance in cats. While other investigators have found that damage to or suppression of the PPC alters bodily awareness in the upper body (Wolpert et al., 1998) or during postural control (Young et al., 2020), this group has extended these findings to locomotor control and suggest that the PPC is involved in the maintenance of a body schema, representing the position of and movement of the limb in space concerning each other and the immediate external surroundings (McVea et al., 2009; Lajoie et al., 2010; Pearson and Gramlich, 2010). The findings of this experiment appear to support this notion. While split-belt walking does involve mechanical changes in the requirements for successful walking, there is strong evidence that corrective movements in split-belt walking are the result of sensorimotor recalibration, not simple mechanical reaction (Iturralde and Torres-Oviedo, 2019). There is also evidence that PPC is involved in other forms of motor adaptation (Mutha et al., 2011; Young et al., 2020). Based on these foundations, we sought to identify the role of the PPC in gait adaptation. To achieve this, three conditions of tDCS stimulation were applied bilaterally to this area before the performance of split-belt adaptation protocols by young and healthy subjects. Additionally, we compared the number of steps required to reach “adapted” walking. We also compared the magnitude of error (i.e., step length symmetry ratio) during Early and Late periods of split-belt walking as well as during the after-effect period.

Comparing between conditions for outcome measures obtained from split-belt walking identified that the number of steps required to adapt to split-belt walking was significantly greater following RA-LC stimulation than Sham stimulation (RA-LC 118.18 ± 104.93, Sham 47.44 ± 52.02). This is noteworthy because RA-LC provided cathodal stimulation to the hemisphere contralateral to the fast leg, suggesting that this stimulation may have impaired sensorimotor adaptation of the last leg. We observed this despite similar magnitude between conditions for step length symmetry ratio at the Early Split and Late Split periods (RA-LC Early Split 0.49 ± 0.23, RA-LC Late Split 0.22 ± 0.13, Sham Early Split 0.34 ± 0.19, Sham Late Split 0.24 ± 0.04). This finding suggests that all conditions experienced the same initial perturbation and were able to adapt to split-belt walking to a similar extent after 15 min, but the time-course needed to adapt was altered by stimulation. Therefore, the time-course necessary to adapt to split-belt walking is increased following RA-LC stimulation, which results in inhibition of the left PPC and excitation of the right PPC. This may suggest that the left PPC is critically involved in locomotor adaptability, however, the bilateral nature of this stimulation makes it impossible to fully delineate the contributions of the left and right hemisphere PPC. These results are generally supportive of previous studies that have investigated the role of the PPC in upper body motor adaptation. Desmurget et al. (1999) identified that modulation of the PPC can disrupt online adaptation to correct erroneous hand trajectories in reaching tasks. These results were later confirmed and extended by Della-Maggiore et al. (2004). Magnani et al. (2013) identified that inhibitory stimulation of the left PPC but not right PPC altered prism adaptation, suggesting a direct involvement of the left PPC on sensory and spatial modulations brought on by prism adaptation. Mutha et al. (2011) identified that patients with lesions of the right parietal cortex exhibited normal adaptation and after-effects during and after a visuomotor adaptation task, while patients with left parietal damage showed decreased adaptation and absent after-effects. The authors demonstrated the importance of the left hemisphere parietal cortex in visuomotor adaptation. The current investigation extended these findings to the realm of gait adaptation. Our results generally reinforce those of the aforementioned articles and identify that inhibition of the left PPC alters motor adaptation.

These results are also somewhat in concert with our previous findings that active stimulation of the PPC decreased postural adaptation (Young et al., 2020). Both experiments found that RA-LC stimulation altered adaptation, however, the aforementioned also found altered adaptation following RC-LA stimulation. This investigation observed slight trends in that direction; however, the results did not meet or approach statistical significance. The different outcomes between our two experiments may be due to the differences in the task. While our postural adaptation task was symmetrical, split-belt treadmill paradigms are inherently asymmetrical.

As the brain is highly interconnected and most processes are handled by a diffuse network of structures, the PPC is not the only region indicated in motor adaptation. In their 2012 publication, Jayaram et al. (2012) utilized tDCS of the cerebellum to investigate adaptation to a similar split-belt walking intervention. They identified that anodal stimulation of the cerebellum improved adaptation of spatial parameters while cathodal tDCS impaired adaptation rate (Jayaram et al., 2012). The group did not observe any differences in the magnitude of after-effects or the rate of de-adaptation. Similar to Jayaram et al. (2012), we identified that stimulation altered the time-course of adaptation to split-belt walking, but not the magnitude of asymmetry (i.e., step length symmetry ratio) during the Early Split or Late Split periods. Conversely, unlike Jayaram et al. (2012), in our study, the after-effects of split-belt exhibited different responses. The number of steps needed to re-adapt to tied-belt walking was not dependent on stimulation condition (Sham 22.76 ± 20.48, RA-LC 18.49 ± 20.33, RC-LA 17.39 ± 17.91); however, the magnitude of step length symmetry ratio was affected. RA-LC stimulation led to greater after-effects throughout the after-effect period (RA-LC Early Tied −0.27 ± 0.24, RA-LC Late Tied −0.06 ± 0.07, Sham Early Tied −0.09 ± 0.19, Sham Late Tied −0.04 ± 0.04). These results show that inhibition of the left PPC decreases the ability to resolve after-effect (i.e., errors). The lack of significant effects of condition on the decay constant during the after-effect period may not inherently reflect physiological differences. Because step length symmetry ratio during Early Tied and Late Tied after-effect periods were greater following RA-LC, the step length symmetry asymptote may have also been greater. These data suggest that the PPC may not be involved in the rate of re-adaptation to original conditions but is involved in the magnitude of after-effects. Together, our results, coupled with those of Jayaram et al. (2012), reinforce the knowledge that multiple brain regions are involved in adaptation. There are reciprocal connections between the PPC and cerebellum (Amino et al., 2001; Clower et al., 2001). These connections allow the PPC to utilize the efferent copy, provided by the cerebellum, and sensory information which the PPC integrates, to maintain and update an internal representation, allowing adaptation (Amino et al., 2001; Parkinson et al., 2010). As tDCS in this study affected PPC excitability, it likely also altered the functionality of the reciprocal connections between the PPC and the cerebellum. It is also possible that networks including the PPC and other cortical or subcortical areas were altered as a result of this stimulation. The results of this study reinforce the need to understand these networks and complex processes.

We know that adaptability to a split-belt treadmill walking paradigm may provide insights as to whether or not a patient retains enough plasticity to be successfully rehabilitated following a stroke (Bastian, 2008). Furthermore, in stroke patients, a bout of split-belt walking has been shown to acutely improve gait symmetry (Reisman et al., 2007). It has also been shown that a single exposure to other adaptation paradigms can lead to increased performance for as long as 5 days in stroke (Rossetti et al., 1998; Pisella et al., 2002). Furthermore, in some patients, repeated exposure to split-belt walking may lead to well retained improved gait performance post-stroke (Reisman et al., 2013). Therefore, it may be worthwhile to explore whether nor not improving the excitability of the contralesional PPC through non-invasive brain stimulation, paired with a split-belt walking training intervention is capable of improving gait long-term in patients with stroke. While this study only observed impaired adaptation following RA-LC stimulation, and not facilitated adaptation following RC-LA stimulation, our subject population of healthy young adults could have contributed to a ceiling effect. More research in diverse subject populations is needed to identify if there is an effect of excitatory stimulation of the PPC on locomotor adaptation.

Beyond rehabilitation, in the realm of space-flight, it has been suggested that endeavors to improve adaptability pre-flight may facilitate the individual’s ability to “learn how to learn,” and improve their general adaptability to more successfully re-orient to the earth upon return (Seidler, 2010; Bloomberg et al., 2015). Developing new adaptation paradigms and batteries, as well as developing new tools to maximize their productivity is therefore of value. Possibly the scientists could utilize neuromodulation of the PPC in such a way that it improves the person’s ability to “learn how to learn.”

This study stimulated the bilateral PPC instead of placing the return electrode on another brain region, such as the supraorbital foramen, like other studies have done (Ishigaki et al., 2016). This was performed to avoid stimulation of other brain regions, such as the motor cortices. Because of this, as one hemisphere received inhibitory stimulation, the other received excitatory stimulation. Based on this choice, perhaps our stimulation paradigm resulted in intra-hemispheric interactions that are more complicated than can be described simply by the sum of anodal and cathodal stimulation (Lindenberg et al., 2013). There is also evidence that bilateral tDCS may result in inter-hemispheric imbalances in excitability (Sehm et al., 2013). Due to the bilateral stimulation paradigm, and inherently asymmetry in the task of split-belt walking, it is impossible to make strong conclusions regarding hemisphere specific contributions to motor adaptation. Furthermore, due to the utilization of a sample comprised solely of young and healthy individuals, and the level of inter-subject variability, further research is necessary before generalization of these results is possible. Nevertheless, the current results do suggest that PPC is involved in the process of gait adaptation. It is most likely that this involvement is through sensory integration, and that decreased adaptability following inhibition of the left PPC is due to impaired sensory integration, leading to decreased efficiency of the identification and rectification of error through a sensorimotor recalibration and implicit learning (Leech and Roemmich, 2018; Young et al., 2020). Future studies should extend and refine these findings by using techniques such as repetitive transcranial magnetic stimulation to achieve more focal stimulation. A previous publication by Johannsen et al. (2014) disrupted the left inferior parietal gyrus before a sensory-integration-based postural task, verifying the potential for repetitive transcranial magnetic stimulation over parietal structures in lower body motor control. This basic protocol could be effectively generalized to other adaptation paradigms. Future studies should also endeavor to utilize neuroimaging to correlate acute cortical changes with locomotor adaptability.

## Conclusions

To identify the role of the PPC in gait adaptation, three bouts of tDCS were applied in a randomized, double-blind fashion. Following each bout, a split-belt adaptation protocol was performed. We identified a greater number of steps required to adapt following RA-LC stimulation. We also found a greater magnitude of after-effects following RA-LC stimulation. Together these results suggest that the PPC is involved in locomotor adaptation. Future research should include brain imaging to correlate locomotor adaptation to activity in the PPC and should include clinical populations to identify the efficacy of tDCS of the PPC on improving locomotor function.

## Data Availability Statement

The raw data supporting the conclusions of this article will be made available by the authors, without undue reservation.

## Ethics Statement

The studies involving human participants were reviewed and approved by University of Houston Institutional Review Board for Experimental Studies. The patients/participants provided their written informed consent to participate in this study.

## Author Contributions

DY, PP, and CL contributed to the study design. DY recruited the subjects, was involved in the informed consent process, processed, analyzed data, and wrote the first draft of the manuscript. All authors contributed to data interpretation and manuscript revision. All authors contributed to the article and approved the submitted version.

## Conflict of Interest

The authors declare that the research was conducted in the absence of any commercial or financial relationships that could be construed as a potential conflict of interest.
